# Digitizing Micromaser Steady States: Entropy, Information Graphs, and Multipartite Correlations in Qubit Registers

**DOI:** 10.3390/e28020162

**Published:** 2026-01-31

**Authors:** István Németh, Szilárd Zsóka, Attila Bencze

**Affiliations:** Kandó Kálmán Faculty of Electrical Engineering, Óbuda University, 1034 Budapest, Hungary; zsoka.szilard@kvk.uni-obuda.hu (S.Z.); bencze.attila@kvk.uni-obuda.hu (A.B.)

**Keywords:** von Neumann entropy, per-qubit entropies, negativity, three-tangle, multipartite entanglement, qubit register embedding

## Abstract

We develop a digitization-based analysis workflow for characterizing the entropy and correlation structure of truncated bosonic quantum fields after embedding them into small qubit registers, and illustrate it on the steady state of a coherently pumped micromaser. The cavity field is truncated to 32 Fock levels and embedded into a five-qubit register via a Gray-code mapping of photon number to computational basis states, with binary encoding used as a benchmark. On this register we compute reduced entropies, mutual informations, bipartite negativities and Coffman–Kundu–Wootters three-tangles for all qubit pairs and triplets, and use the resulting patterns to define information graphs. The micromaser Liouvillian naturally supports trapping manifolds in Fock space, whose structure depends on the choice of interaction angle and on thermal coupling to the reservoir. We show that these manifolds leave a clear imprint on the digitized information graph: multi-block trapping configurations induce sparse, banded patterns dominated by a few two-qubit links, while trapping on a single 32-dimensional manifold or coupling to a thermally populated cavity leads to more delocalized and collectively shared correlations. The entropy and mutual-information profiles of the register provide a complementary view on how energy and information are distributed across qubits in different parameter regimes. Although the full micromaser dynamics can in principle generate higher-order entanglement, we focus here on well-defined measures of two- and three-party correlations and treat the emerging information graph as a structural probe of digitized field states. We expect the workflow to transfer to other bosonic fields encoded in small qubit registers, and outline how the resulting information-graph view can serve as a practical diagnostic in studies of driven-dissipative correlation structure.

## 1. Introduction

Driven-dissipative cavity QED systems are paradigmatic platforms for studying how nonclassical steady states arise from the competition between coherent drive and environmental noise. The micromaser, in which a stream of two-level atoms traverses a single-mode cavity coupled to a thermal reservoir, is a particularly well-controlled example. Its steady-state photon statistics, including trapping states and nonclassical features of the cavity field, have been characterized in detail in terms of Fock-space dynamics and effective Liouvillians [1,2,3,4,5,6,7].

Phase properties of the micromaser field have also been extensively analyzed in the Pegg–Barnett phase formalism and related approaches, revealing nontrivial phase distributions and phase transitions in the steady state [8,9,10,11,12]. In the present work we focus on Fock-space digitization and information graphs; a detailed phase-basis digitization in the Pegg–Barnett sense is left for future studies.

From a quantum information perspective, the micromaser has been studied as a channel for information transfer and entanglement generation between atoms and the field [13] and as a platform where cavity fields mediate correlations between successive atomic probes. Related questions about transferring entanglement between bosonic modes and qubits have been explored in continuous-variable to qubit interfaces [14,15,16].

In many quantum information and quantum simulation settings, however, one is ultimately interested in finite qubit registers rather than in continuous or high-dimensional bosonic degrees of freedom. This raises a natural question: how should one digitize a bosonic field into a finite number of qubits, and what does the resulting multipartite correlation structure look like in the qubit language? Equivalently, given a fixed truncation of the field Hilbert space, how does the underlying Liouvillian geometry (e.g., trapping manifolds, thermal connectivity) translate into patterns of entanglement and information flow on a qubit register? Recent work on bosonic field digitization for quantum computing highlights similar issues in the context of scalar fields and lattice gauge theories [17,18,19].

Related thermalization questions in multimode nonlinear optical systems and in non-Markovian environments have recently attracted substantial attention (see, e.g., [20,21,22,23,24]). The aim of the present work is not to model such multimode or non-Markovian scenarios, but rather to use an exactly solvable single-mode micromaser as a controlled testbed in which the connection between Liouvillian connectivity (trapping manifolds and thermal bridging) and digitized qubit-level correlation topology can be studied transparently.

Why digitize at all, instead of analyzing the field directly in Fock space? Once a truncation fixes a finite Hilbert space, an encoding into qubits introduces a tensor-product structure that enables subsystem entropies, pairwise negativities, and multipartite measures, i.e., an information-graph view that is not available at the single-mode level. This logical layer is explicitly encoding-dependent; in this work we exploit that dependence (Gray versus binary) as a diagnostic of how Liouvillian connectivity distributes coherence across the Fock ladder and how such coherence appears as qubit correlations.

In this work we address these questions for the steady state of a coherently pumped micromaser. We truncate the cavity field to the lowest 32 Fock levels and embed this 32-dimensional space into a five-qubit register using both Gray and binary encodings of the photon number. On the resulting register we compute reduced von Neumann entropies for all subsystems of interest, mutual informations on selected bipartitions, bipartite negativities for all qubit pairs, and three-tangles for all qubit triplets, and we organize these quantities into information graphs. This provides an explicit and flexible “digitization microscope” for the field state: by working in the qubit basis one can directly inspect which subsets of qubits carry the entropy, mutual information, and entanglement generated by the micromaser dynamics.

A key role is played by the structure of trapping manifolds. For special choices of the atom–field interaction angle, the micromaser Liouvillian partitions Fock space into invariant manifolds, or trapping blocks, inside which dynamics is confined [1]. When the trapping dimension is commensurate with the truncation, the entire 32-dimensional space can be treated as a single trapping block or, for other parameter choices, as several smaller blocks connected only weakly by thermal processes. We show that these manifolds imprint themselves on the digitized information graph in a transparent way, particularly in Gray encoding where photon-number transitions correspond to local bit flips. By comparing different trapping and thermal configurations, we outline a taxonomy of information-graph structures that can arise in the digitized micromaser, without aiming at a complete classification.

We deliberately choose the coherently pumped micromaser as a testbed because its Liouvillian supports analytically controllable trapping manifolds (tunable via *q* and Θ), providing a clean knob to change the effective connectivity of Fock space. By contrast, digitizing an arbitrary high-dimensional state would not provide a meaningful notion of “Liouvillian geometry” nor a physical relation between control parameters and information-graph topology. The analytic steady-state solution of Refs. [1,25] enables systematic parameter sweeps that expose robust, regime-level patterns rather than isolated examples.

The entropy perspective enters in two ways. First, reduced entropy and mutual information of the qubit subsystems provide a complementary view to entanglement monotones, especially when the system is strongly mixed [26,27]. Second, the micromaser is a natural setting in which to discuss energy and information channels on an equal footing: atomic inversion, coherent pump, and thermal occupation control both the steady-state photon number and the amount of coherent structure imprinted on the field. Our results highlight how different Liouvillian geometries distribute entropy and correlations across the register in qualitatively distinct ways.

The present work is complementary to our earlier micromaser analysis in Refs. [1,25], where the emphasis was on analytic steady-state solutions and trapping in Fock space. Specifically, we borrow from those works the analytic construction of the steady-state density matrix ${\hat{\rho }}_{F}^{\left(\infty \right)}$ and the trapping mechanism that partitions Fock space into invariant manifolds. What is new here is the digitization analysis itself: the systematic embedding of the truncated field into a five-qubit register, the Gray-versus-binary encoding comparison, and the resulting entropy/entanglement and information-graph taxonomy across distinct Liouvillian geometries. We structure our analysis around three representative Liouvillian geometries—segmented multi-block trapping, single-block trapping, and a strongly thermally connected hot cavity—and compare how each is reflected in the digitized information graph. Our main contributions are the following:

- We provide a systematic analysis of micromaser steady states viewed as information graphs on a digitized five-qubit register, combining per-qubit entropies, mutual informations, bipartite negativities, and three-tangles over all qubit pairs and triplets.
- We identify three qualitatively distinct Liouvillian geometries (multi-block trapping, single-block trapping, thermally connected hot cavity) and show that each regime has a characteristic entropy and correlation fingerprint on the register, including different topologies of the information graph and three-tangle distribution.
- We demonstrate that in a multi-block trapping regime under weak thermal bridging, the maximum three-tangle remains comparatively robust in parameter ranges where the maximum pairwise negativity collapses, and that in hot-cavity regimes $\tau_{3}^{\max}$ systematically exceeds $N_{\max}$ across a wide range of thermal occupations, providing concrete evidence that Liouvillian geometry can be used as an “entanglement-engineering knob” for digitized bosonic fields.
- We highlight a geometry-aware advantage of Gray encoding for truncated bosonic fields in this micromaser setting: because nearest-neighbor steps on the Fock ladder map to single-bit flips on the Gray-encoded register, trapping manifolds and thermal bridges translates into structurally local features on the information graph, and we compare this directly with the corresponding binary-encoded graphs for the same steady states.

The paper is organized as follows. In Section 2 we briefly review the micromaser model and the trapping mechanism. Section 3 introduces the digitization of the truncated field into a five-qubit register and defines the information graph and analysis framework. In Section 4 we describe the entropic and entanglement measures used in this work, with an emphasis on negativity and three-tangle. Section 5 explains how trapping manifolds and thermal bridging shape the Liouvillian geometry in Fock space. In Section 6 we present representative case studies that illustrate how different parameter regimes lead to distinct information graphs and entropy profiles on the register. We conclude in Section 7 with a discussion of entanglement-engineering perspectives and possible generalizations to other bosonic systems.

## 2. Coherently Pumped Micromaser and Trapping

We consider a single-mode cavity of frequency $\omega_{0}$ interacting with a stream of two-level atoms with atomic transition frequency $\omega_{ab}$ driven before entering the cavity and coupled to a thermal reservoir with mean photon number $n_{\mathrm{th}}$. In a suitable rotating frame the free cavity Hamiltonian is ${\hat{H}}_{F}=\hbar \omega_{0}\;{\hat{a}}^{†}\hat{a}$ and each atom is prepared in a mixed but coherent state

(1) $${\hat{\rho }}_{\mathrm{at}}=\left(\begin{array}{cc} {\hat{\rho }}_{aa} & \lambda \sqrt{{\hat{\rho }}_{aa}{\hat{\rho }}_{bb}}\\ \lambda \sqrt{{\hat{\rho }}_{aa}{\hat{\rho }}_{bb}} & {\hat{\rho }}_{bb} \end{array}\right),\;{\hat{\rho }}_{aa}+{\hat{\rho }}_{bb}=1,$$

with population inversion $u={\hat{\rho }}_{aa}-{\hat{\rho }}_{bb}$ and coherence parameter 0≤λ≤1. Atoms cross the cavity at rate *r*, and we use $N_{\mathrm{ex}}=r/\Gamma$ (the ratio of atomic flux to cavity decay rate) as a dimensionless pump parameter.

The interaction between the atom and the cavity mode during the transit time is described by a Jaynes–Cummings Hamiltonian,

(2) $${\hat{H}}_{\mathrm{JC}}=\hbar g\left(\hat{a}|a\rangle \;\langle b|+{\hat{a}}^{†}|b\rangle \;\langle a|\right)+\hbar \Delta \;|a\rangle \;\langle a|,$$

with coupling strength *g* and detuning $\Delta =(\omega_{0}-\omega_{ab})/\Gamma$. For a fixed transit time τ the JC evolution is characterized by an effective interaction angle Θ=gτ. Between atoms the cavity field relaxes towards a thermal state at rate Γ.

In the limit of at most one atom in the cavity at a time and neglecting atomic spontaneous emission inside the cavity, the dynamics of the cavity state ${\hat{\rho }}_{F}$ is described by a master equation of the form

(3) $${\dot{\hat{\rho }}}_{F}=L_{\mathrm{th}}\left[{\hat{\rho }}_{F}\right]+r\left(E\left[{\hat{\rho }}_{F}\right]-{\hat{\rho }}_{F}\right),$$

where $L_{\mathrm{th}}$ is the thermal Liouvillian and E is the completely positive map induced by one atom interacting with the field via ${\hat{H}}_{\mathrm{JC}}$. The stationary state ${\hat{\rho }}_{F}^{\left(\infty \right)}$ is the fixed point of this micromaser master equation.

Throughout this work we follow the coherently pumped micromaser model and parameterization introduced in Ref. [1] and further summarized in Ref. [25], building on the general micromaser theory of Refs. [4,5,6,7] and related coherent-pumping schemes [2,3]. A detailed derivation of the master equation and its trapping solutions is given in Ref. [1]; here we only summarize the ingredients needed for the subsequent digitization and information-graph analysis.

For special values of the interaction angle, the micromaser exhibits trapping. If Θ is tuned to

(4) $$\Theta =\frac{q\pi }{\sqrt{n_{0}^{2}+\left(n_{q}+1\right)}},\;q=1,2,\ldots ,$$

with an integer $n_{q}$, then population cannot escape beyond photon number $n_{q}$ and the Liouvillian decomposes into invariant subspaces in Fock space. We refer to these invariant subspaces as trapping manifolds or trapping blocks. In Equation (4) $n_{0}=\left(\Gamma /2g\right)\Delta$ is the detuning–index parameter introduced in Ref. [1], which encodes the effective photon number shift due to the detuning of the empty cavity frequency and the atomic transition frequency. Throughout this work we specialize to the resonant case $n_{0}=0$, which is also used in all case studies and figure captions below. Choosing $n_{q}+1$ commensurate with a desired truncation dimension allows one to arrange that the entire truncated Hilbert space is either a single trapping block or subdivided into several blocks whose structure depends on the integer *q*.

Throughout this work we truncate the cavity Hilbert space to $n_{\max}=31$ photons, corresponding to a 32-dimensional space, and consider parameter regimes in which the steady state is well confined within this truncation. We focus on configurations where $n_{q}+1=32$, so that the trapping manifold either coincides with the truncated space or partitions it into several smaller blocks.

For each parameter set we obtain the steady state ${\hat{\rho }}_{F}^{\left(\infty \right)}$ using the analytic method derived in Ref. [1], and then truncate it to $n_{\max}=31$ photons. For all configurations considered here, the population above n=31 remained below $10^{-6}$, so the 32-dimensional truncation provides an accurate representation of the steady-state field.

The resulting truncated field steady state provides the input for the digitization step. In the next section we impose a qubit tensor-product structure on $H_{F}^{\left(32\right)}$ and evaluate subsystem-resolved information measures on the resulting register state.

## 3. Digitization into a Five-Qubit Register

### 3.1. Truncation and Encoding

The truncated field Hilbert space is spanned by the photon-number basis ${\left\{|n\rangle \right\}}_{n=0}^{31}$ and has dimension d=32. We embed this space into a five-qubit register,

(5) $$H_{F}^{\left(32\right)}≅H_{Q}={\left(C^{2}\right)}^{⊗5},$$

by specifying a bijection between photon number and computational basis states. We consider two encodings:

Binary encoding

In the binary encoding,

(6) $$n=\sum_{j=0}^{4}b_{j}2^{j}\;⟷\;|b_{4}b_{3}b_{2}b_{1}b_{0}\rangle ,$$

with $b_{j}\in \left\{0,1\right\}$. This is the standard mapping and is convenient from a classical information perspective.

Gray encoding

In the Gray encoding, on which we will focus, successive photon numbers differ by a single bit flip. Let $b_{j}$ denote the binary bits as above and define Gray bits

(7) $$g_{j}=b_{j}⊕b_{j+1},\;j=0,\ldots ,4,\;b_{5}\equiv 0.$$

The Gray mapping is then

(8) $$n\in \{0,\ldots ,31\}\;⟷\;|g_{4}g_{3}g_{2}g_{1}g_{0}\rangle .$$

This mapping is implemented by a fixed unitary on the truncated field space, so the register density operator ${\hat{\rho }}_{Q}=U_{\mathrm{enc}}{\hat{\rho }}_{F}U_{\mathrm{enc}}^{†}$ is unitarily equivalent to ${\hat{\rho }}_{F}$.

The Gray encoding is geometry-aware: elementary photon-number transitions n→n±1 correspond to single-bit flips on the register, so nearest-neighbor structure in Fock space becomes local structure in qubit space. This will be crucial for interpreting trapping manifolds and thermal bridging in terms of information graphs. In what follows we label the five logical qubits by $b_{0},\ldots ,b_{4}$, regardless of whether they currently store the binary or the Gray-encoded bits; the encoding in use will always be clear from context.

These qubits are *logical subsystems* induced by the chosen tensor-product identification $H_{F}^{\left(32\right)}≅{\left(C^{2}\right)}^{⊗5}$; they are not five physical constituents interacting inside the cavity. Consequently, register entanglement quantifies how coherence and mixing of the single bosonic mode are distributed across this particular qubit factorization. In particular, if ${\hat{\rho }}_{F}$ is diagonal in the photon-number basis then the encoded register state ${\hat{\rho }}_{Q}$ is diagonal in the computational basis for both encodings and all bipartite negativities vanish; nonzero two- and three-party entanglement in the register is therefore a signature of off-diagonal coherence in the truncated field state under the chosen encoding. Intuitively, such register entanglement is generated by coherences between photon-number components whose Gray/binary codewords differ in multiple bit positions, so that a single-mode superposition maps to multipartite register correlations.

### 3.2. Digitization and Information-Graph Framework

Given any truncated bosonic field state ${\hat{\rho }}_{F}$ on a *d*-dimensional Hilbert space with $d=2^{n}$, we analyze its entropy (all entropies are measured in bits throughout this work) and correlation structure after digitization into an *n*-qubit register in a model-independent way. The procedure consists of the following steps:

(i) *Encoding and embedding.* Choose a qubit encoding (binary, Gray, or another bijection) and construct a fixed unitary $U_{\mathrm{enc}}:H_{F}^{\left(d\right)}\to {\left(C^{2}\right)}^{⊗n}$. The digitized register state is then
  (9) $${\hat{\rho }}_{Q}=U_{\mathrm{enc}}{\hat{\rho }}_{F}U_{\mathrm{enc}}^{†},$$
  which is unitarily equivalent to ${\hat{\rho }}_{F}$.
(ii) *Reduced states.* For all relevant subsets *A* of qubits (single sites, pairs, triplets and, if needed, larger subsets), compute the reduced density operators by partial tracing:
  (10) $${\hat{\rho }}_{A}={\mathrm{Tr}}_{\bar{A}}{\hat{\rho }}_{Q}.$$
(iii) *Entropic and entanglement diagnostics.* From the reduced states, evaluate
  - Single-qubit von Neumann entropies $S\left(b_{i}\right)=-\mathrm{Tr}\left({\hat{\rho }}_{b_{i}}\log_{2}{\hat{\rho }}_{b_{i}}\right)$;
  - Mutual informations I(A:B)=S(A)+S(B)−S(AB) between chosen subsystems;
  - Bipartite negativities $N(b_{i}:b_{j})$ for all qubit pairs;
  - Three-tangles $\tau_{3}\left(b_{i}:b_{j}:b_{k}\right)$ for all qubit triplets.
(iv) *Information graph and triplet map.* Interpret the *n* qubits as nodes of an information network with
  - Node attributes given by $S(b_{i})$ and local purities;
  - Edge weights $w_{ij}=N\left(b_{i}:b_{j}\right)$, defining the *information graph*;
  - Triplet attributes $\tau_{3}\left(b_{i}:b_{j}:b_{k}\right)$, providing a map of genuine three-party correlations across all n3 triplets.
  In the visualizations used below, we supplement these edge weights with mutual-information data: edge thickness is chosen proportional to $I(b_{i}:b_{j})$, while negativity $N(b_{i}:b_{j})$ is encoded by edge gray level and also reported in the edge labels together with the corresponding mutual information.
(v) *Comparison across encodings and regimes.* By repeating steps (i)–(iv) for different encodings or different dynamical regimes of the underlying bosonic model, one can compare how Liouvillian geometry, truncation, and encoding choice are reflected in the resulting information graphs and triplet maps.

This workflow is independent of the specific physical origin of ${\hat{\rho }}_{F}$ and applies to any truncated bosonic mode encoded into a finite qubit register. In the present micromaser application, we implement it for d=32 and n=5 in the Fock basis. For every parameter set, we first compute the truncated steady-state field ${\hat{\rho }}_{F}$ and then construct two digitized five-qubit states by applying the appropriate encoding unitaries, one for the binary mapping and one for the Gray mapping. In each encoding we recompute all reduced density operators and evaluate the corresponding quantities separately: global and single-qubit entropies, mutual informations, bipartite negativities, and, wherever well-defined, three-tangles on all qubit triplets. Thus, every data point in our database has a binary-encoded and a Gray-encoded version derived from the same underlying field state. Throughout this paper we focus on the Gray-encoded Fock basis in the figures and tables, because it makes the connection between Fock-space geometry (trapping manifolds and thermal bridging) and local structure on the qubit register the most transparent; for selected configurations we also show the corresponding binary-encoded information graphs for direct comparison.

**Figure 1:**
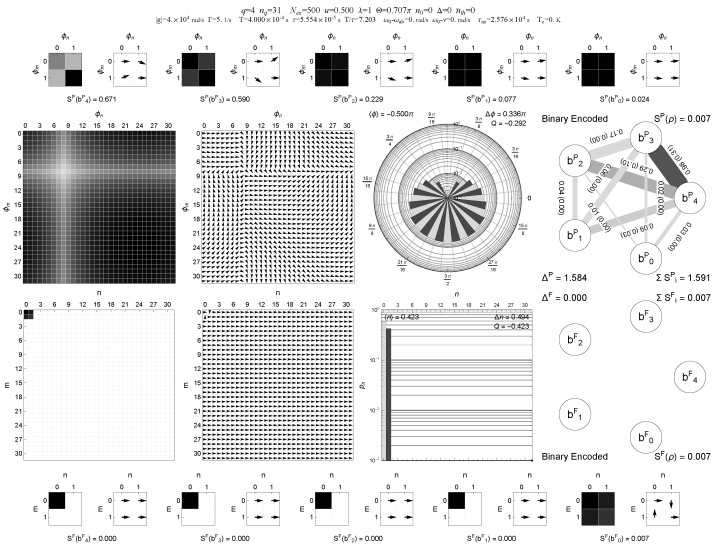
Multi-panel “QubitInfoPanel” visualization of the micromaser steady state for a multi-block trapping configuration in **binary encoding**, at zero thermal occupation (q=4, $n_{q}+1=32$, $N_{\mathrm{ex}}=500$, u=0.5, λ=1, $n_{\mathrm{th}}=0$, $n_{0}=0$, Δ=0, trapping value of Θ with $n_{q}+1=32$). The middle block of panels shows the steady-state field density matrix in two representations. In the upper middle row the state is displayed in the Pegg–Barnett phase basis (magnitude and argument), together with the phase distribution and Mandel parameter, providing a visual impression of the phase structure of the steady state. In the lower middle row, the same steady state is shown in the Fock basis (density plot and argument), together with the photon-number distribution and Mandel parameter. To the right of these field plots, we display the corresponding information graphs in the respective bases. In each information graph, nodes represent the five qubits and edges represent pairwise correlations. Edge thickness encodes the mutual information $I(b_{i}:b_{j})$, edge gray level encodes the bipartite negativity $N(b_{i}:b_{j})$, and each edge is labeled by $I(b_{i}:b_{j})$ with $N(b_{i}:b_{j})$ given in parentheses. The outer rows (top and bottom) summarize the digitized five-qubit register in the respective basis and in binary encoding, collecting the single-qubit entropies and other register-level diagnostics for the same steady state. Central insight: in the cold multi-block trapping regime, correlations are strongly structured and encoding-dependent; the binary graph provides a benchmark for the Gray-local picture in Figure 3.

**Figure 2:**
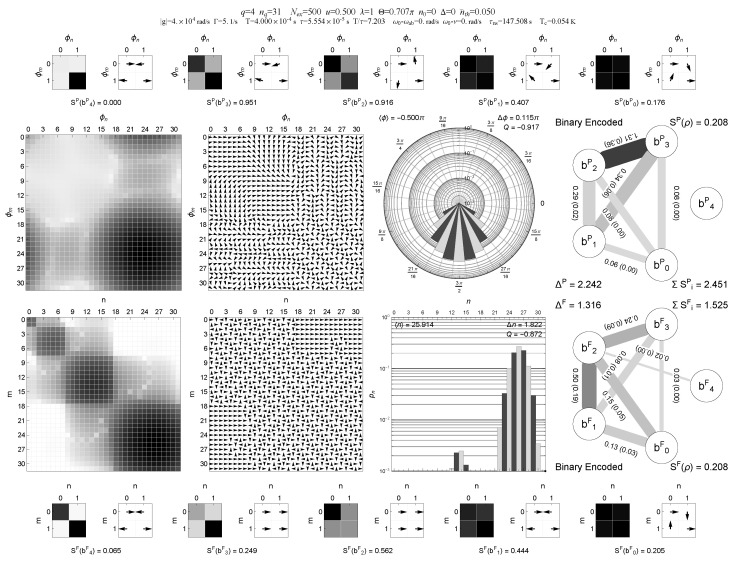
Same configuration and multi-panel layout as in Figure 1, but with a small thermal population $n_{\mathrm{th}}=0.05$. The phase- and Fock-basis density-matrix panels show how thermal bridging starts to populate additional Fock states and smear out phase coherences. On the digitized binary-encoded register, the information graph reveals the qualitative effect discussed in Section 6: the strongest two-qubit links, as measured by negativity, weaken dramatically once $n_{\mathrm{th}}>0$, while the overall pattern of mutual informations and node entropies becomes more homogeneous. Together with Figure 5, these panels visualize how even a very small thermal occupation reshapes the correlation structure induced by multi-block trapping. Central insight: even weak thermal occupation reshapes the correlation topology, weakening trapping-induced structure and redistributing correlations across the register.

**Figure 3:**
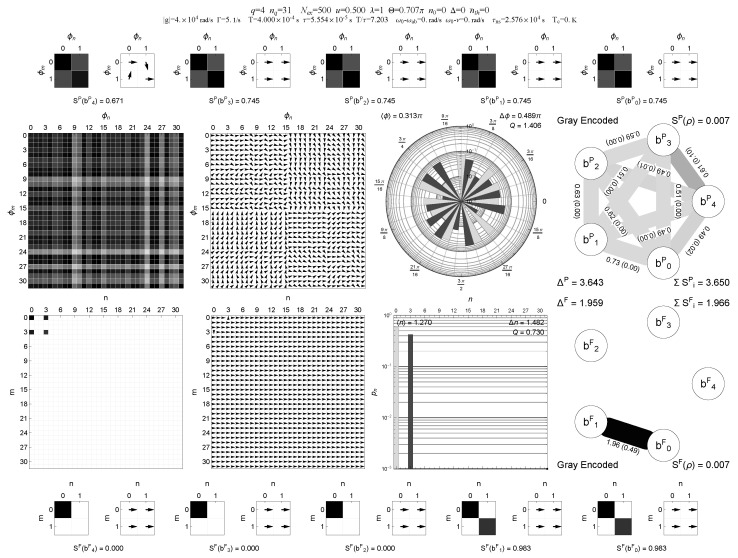
Multi-panel “QubitInfoPanelGray” visualization of the same multi-block trapping steady state as in Figure 1, but now digitized in **Gray encoding**. The middle panels again show the field density matrix in the Pegg–Barnett phase basis and in the Fock basis (magnitude and phase), together with the phase and photon-number distribution. In Gray encoding, nearest-neighbor photon-number transitions n→n±1 map to single-bit flips on the qubit register, so the trapping manifolds appear as structurally local features. This is reflected in the lower information-graph panel: entropy is concentrated on a subset of Gray bits, and a small number of edges carry most of the bipartite negativity, with edge labels listing the mutual information and negativity for each qubit pair. The Gray-encoded information graph thus emphasizes the direct connection between Liouvillian trapping blocks in Fock space and local correlation structure on the qubit register. Central insight: Gray encoding maps Fock-ladder locality to local bit flips, making trapping-manifold structure appear as localized entropy and a few dominant register links.

**Figure 4:**
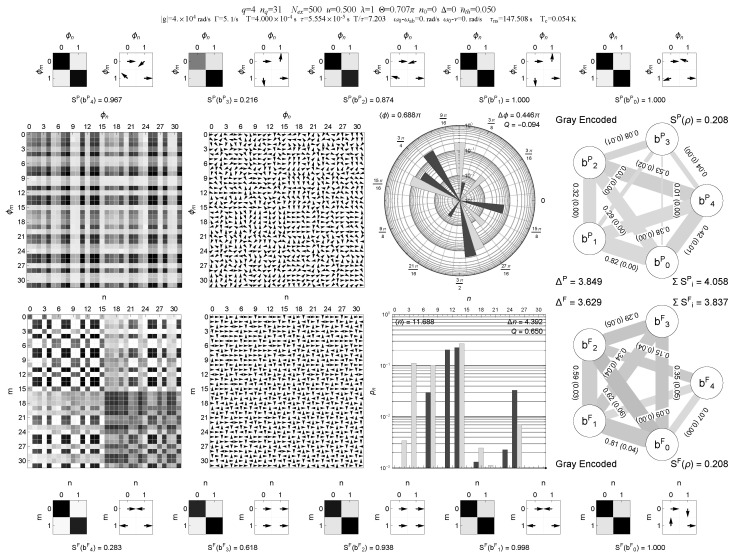
Same Gray-encoded multi-panel visualization as in Figure 3, but with a small thermal photon number $n_{\mathrm{th}}=0.05$. The field-level panels show the onset of thermal bridging between trapping blocks in both phase and Fock representations, with weight accumulating near block boundaries. On the five-qubit Gray register, the information graph exhibits the qualitative change quantified in Figure 5: the dominant two-qubit negativity links are strongly suppressed, while node entropies increase and mutual information becomes more broadly distributed over the register. These Gray-encoded panels make the interplay between trapping geometry, thermal bridging, and digitized information-graph topology directly visible in a single figure. Central insight: with $n_{\mathrm{th}}>0$, Gray-local dominant links are strongly suppressed and correlations become more broadly distributed across the register.

A representative comparison between binary and Gray encodings for the same multi-block trapping configuration (case A) is shown in Figure 1, Figure 2, Figure 3 and Figure 4, where the information graphs, node entropies and mutual informations follow similar qualitative trends in both encodings, while the Gray representation makes the connection between trapping manifolds and local qubit structure more transparent.

**Figure 5:**
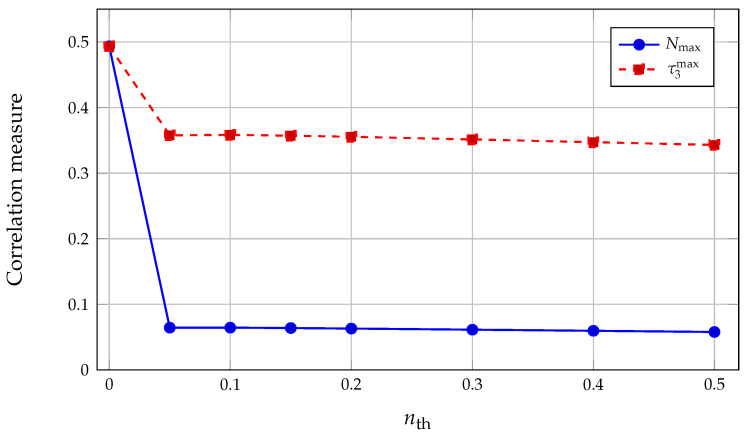
Maximum pairwise negativity $N_{\max}$ and maximum three-tangle $\tau_{3}^{\max}$ in the Gray-encoded five-qubit register as a function of thermal photon number $n_{\mathrm{th}}$ for an inverted-atom, multi-block trapping configuration (q=4, $N_{\mathrm{ex}}=500$, u=0.5, λ=1, $n_{0}=0$, Δ=0). Here, $\tau_{3}^{\max}$ is taken as the maximum of $\tau_{3}$ over those qubit triplets whose reduced three-qubit states are effectively pure according to the eigenvalue-based gate described in Section 4. At $n_{\mathrm{th}}=0$ the register is almost pure and both $N_{\max}$ and $\tau_{3}^{\max}$ are large and equal. As soon as a small thermal population is introduced, $N_{\max}$ collapses by almost an order of magnitude, while $\tau_{3}^{\max}$ remains comparatively robust, so that $\tau_{3}^{\max}/N_{\max}$ increases sharply when thermal bridging between trapping blocks is activated.

## 4. Entropy and Correlation Measures

### 4.1. Entropic Quantities

For any subset *A* of qubits, we define the reduced state ${\hat{\rho }}_{A}={\mathrm{Tr}}_{\bar{A}}{\hat{\rho }}_{Q}$ and its von Neumann entropy

(11) $$S\left(A\right)=-\mathrm{Tr}\left({\hat{\rho }}_{A}\log_{2}{\hat{\rho }}_{A}\right),$$

which we use to construct information-theoretic quantities such as mutual information and conditional entropy [26]. From these we construct the mutual information between two subsystems,

(12) I(A:B)=S(A)+S(B)−S(AB),

and, if needed, conditional entropies and higher-order entropy combinations. Single-qubit entropies characterize local mixedness; mutual informations reflect both classical and quantum correlations between subsystems.

### 4.2. Bipartite Negativity

To quantify bipartite entanglement between two qubits $b_{i}$ and $b_{j}$, we use the negativity [27,28]. Given a two-qubit state ${\hat{\rho }}_{ij}$, we define the partial transpose with respect to party *j* and compute the trace norm of ${\hat{\rho }}_{ij}^{T_{j}}$. The negativity is

(13) $$N\left(b_{i}:b_{j}\right)=\frac{∥{\hat{\rho }}_{ij}^{T_{j}}{∥}_{1}-1}{2}.$$

Negativity is an entanglement monotone for bipartite systems and is sensitive to all two-qubit entanglement present in the register.

### 4.3. Three-Tangle and Multipartite Structure Beyond Three Parties

**Figure 6:**
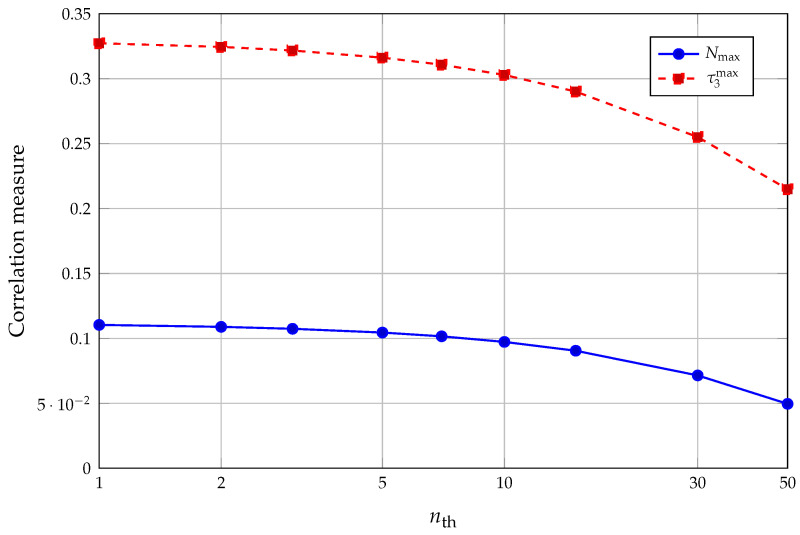
Maximum pairwise negativity $N_{\max}$ and maximum three-tangle $\tau_{3}^{\max}$ in the Gray-encoded five-qubit register as a function of thermal photon number $n_{\mathrm{th}}$ for a hot-cavity regime with near-ground atoms (nontrapping label q=0, $N_{\mathrm{ex}}=2250$, u=−0.5, λ=0.2, $n_{0}=0$, Δ=0, Θ≈0.30). As in Figure 5, $\tau_{3}^{\max}$ is defined as the maximum of $\tau_{3}$ over those qubit triplets whose reduced three-qubit states are effectively pure according to the eigenvalue-based gate specified in Section 4. Both measures decrease as the cavity becomes more thermal, but $\tau_{3}^{\max}$ stays significantly larger than $N_{\max}$ over the whole range, consistent with collectively shared correlations on a highly mixed background.

**Figure 7:**
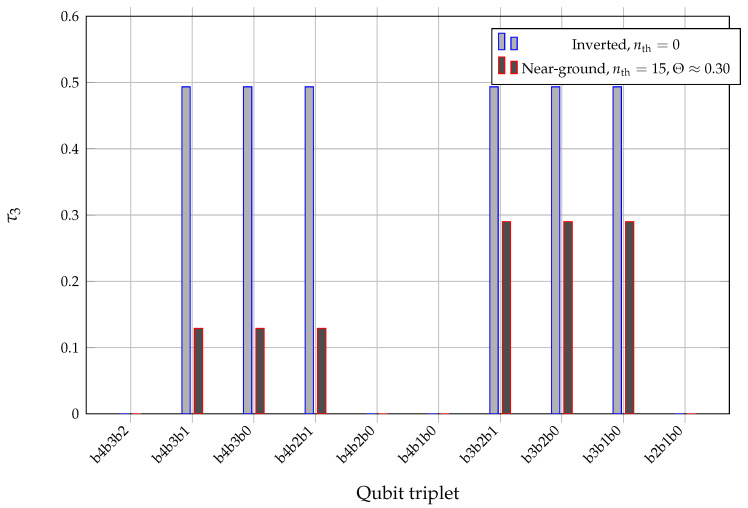
Three-tangle $\tau_{3}$ in the Gray-encoded five-qubit register for all qubit triplets, comparing the multi-block trapping configuration case A (light bars, q=4, $N_{\mathrm{ex}}=500$, u=0.5, $n_{\mathrm{th}}=0$) with the thermally connected hot-cavity configuration case C (dark bars, nontrapping label q=0, $N_{\mathrm{ex}}=2250$, u=−0.5, $n_{\mathrm{th}}=15$, Θ≈0.30). In the inverted, cold case $\tau_{3}$ is more evenly distributed across triplets, whereas in the hot-cavity case it concentrates on an “information core” of low-order Gray bits, illustrating the change in tripartite correlation topology between the two Liouvillian geometries.

For three qubits A,B,C in a pure state, the Coffman–Kundu–Wootters three-tangle $\tau_{3}\left(A:B:C\right)$ provides a measure of genuine tripartite entanglement, satisfying the monogamy relation [29] as follows:

(14) $$C_{A|BC}^{2}=C_{AB}^{2}+C_{AC}^{2}+\tau_{3}\left(A:B:C\right),$$

where *C* denotes concurrence [30]. For mixed three-qubit states one can define $\tau_{3}$ via the convex roof construction over all pure-state decompositions of the density operator, and practical evaluation schemes for this convex roof have been developed in Ref. [31]. In the present work we do not attempt a full convex-roof optimization for arbitrary mixed three-qubit reductions. Instead, for each three-qubit marginal of the five-qubit register, we first test whether it is numerically almost pure: if its largest eigenvalue differs from one by less than a fixed tolerance ($\varepsilon =10^{-10}$ in our numerics) and the remaining eigenvalues are below this threshold, we treat the state as effectively pure and evaluate the Coffman–Kundu–Wootters three-tangle using the standard formula with concurrences obtained from Wootters’ expression for two qubits [29,30]. Triplets that do not pass this purity gate are classified as “three-tangle undefined” in our analysis; in the numerical database they are marked as missing and are excluded from all $\tau_{3}$-based averages, maxima, and statistics. All quoted values of $\tau_{3}$ (such as $\tau_{3}^{\max}$ in Table 1 and in Figure 5, Figure 6 and Figure 7) are thus computed exclusively from those triplets whose reduced states are effectively pure according to this criterion. We verified that varying the threshold ε between $10^{-8}$ and $10^{-12}$ does not change any of the qualitative trends reported in Table 1 and Figure 5 and Figure 6; the value $\varepsilon =10^{-10}$ was chosen as a conservative compromise between numerical precision and robustness. In all parameter regimes considered here, at least one triplet passes this gate, so $\tau_{3}^{\max}$ is always well defined.

The micromaser dynamics acts on an infinite-dimensional Hilbert space and, in principle, can generate entanglement of arbitrarily high order between the cavity mode, the atomic beam, and the reservoir. After truncating the field to 32 Fock levels and embedding it into a five-qubit register, the steady state may still contain genuinely four- and five-partite correlations. However, there is currently no single, universally accepted scalar entanglement monotone for four or more qubits that plays the same role as the three-tangle for three qubits; see, e.g., Refs. [27,31,32] for discussions of convex-roof and monogamy-based approaches.

In this work we therefore restrict our analysis to two well-established quantities: bipartite negativities between all qubit pairs, and the three-tangle on all qubit triplets. Negativity captures two-party entanglement in every two-qubit reduced state ${\hat{\rho }}_{ij}$, while the three-tangle isolates genuine tripartite contributions within each triple. Higher-order contributions are not resolved separately here and are effectively folded into the bipartite and tripartite sharing patterns that we report. The present study should thus be viewed as a controlled lower bound on the available entanglement: any genuinely four- or five-partite correlations may be present in the digitized micromaser steady states, but they are not quantified by an additional scalar monotone in this work.

In the multi-panel visualizations used below, the information graphs encode, for each single-qubit pair $\left(b_{i},b_{j}\right)$, the mutual information $I(b_{i}:b_{j})$ and the bipartite negativity $N(b_{i}:b_{j})$ as follows: edge thickness is proportional to $I(b_{i}:b_{j})$, edge gray level (darker edges) is proportional to $N(b_{i}:b_{j})$, and the numerical edge labels list $I(b_{i}:b_{j})$ with $N(b_{i}:b_{j})$ given in parentheses. This combined representation is used in Figure 1, Figure 2, Figure 3 and Figure 4.

With these subsystem-resolved diagnostics defined, we next summarize how the micromaser Liouvillian organizes number states into trapping manifolds and how thermal activation bridges them. This regime picture guides the case studies and parameter sweeps reported in the following sections.

## 5. Liouvillian Geometry: Trapping Manifolds and Thermal Bridging

The micromaser master equation defines a Liouvillian superoperator on the truncated Fock space. Its structure is conveniently visualized as a graph whose nodes are photon-number states and whose edges represent coherent and incoherent transitions induced by the Jaynes–Cummings interaction and the thermal reservoir [1,4,5].

For generic interaction angles Θ, the JC dynamics couples many photon-number states, and combined with the thermal jumps *a* and $a^{†}$ this yields a highly connected graph. For special values of Θ satisfying the trapping condition (4), however, the Liouvillian decomposes into invariant manifolds. When the trapping dimension $n_{q}+1$ matches the truncation, the 32-dimensional space can be either a single trapping block or partitioned into several smaller blocks.

We distinguish qualitatively between

- *Multi-block trapping geometries*, in which the truncated space splits into several invariant blocks whose sizes and connectivity depend on *q*. At zero thermal occupation, population, and coherence cannot cross between blocks; for finite $n_{\mathrm{th}}$ thermal jumps connect blocks only via a small number of boundary states. We refer to these block-connecting processes as *thermal bridging*.
- *Single-block trapping geometries*, in which the entire 32-dimensional truncated space forms one invariant manifold, even at the trapping condition. Here the JC and thermal transitions act within a single connected block, and thermal coupling can populate a broad band of photon numbers without internal segmentation.
- *Thermally connected geometries*, in which trapping is not enforced and the cavity is significantly populated by the reservoir. In this case the Liouvillian graph is strongly connected and the field state can be viewed as a high-entropy background modified by the repeated action of the atomic map. In our numerical dataset we label such nontrapping configurations by q=0, which is a bookkeeping index and is not intended as a solution of Equation (4).

The central observation of this work is that these Liouvillian geometries have qualitatively different fingerprints on the digitized information graph. Multi-block trapping tends to confine correlations to a few strands in Gray space and promotes sparse, banded patterns of negativity, while single-block trapping and thermally connected geometries facilitate more delocalized correlation structures across the register.

In the next section we illustrate these fingerprints through three representative case studies, chosen to highlight (i) multi-block trapping, (ii) single-block trapping, and (iii) thermal bridging.

## 6. Case Studies: Entropy and Information Graphs

We now illustrate the digitization framework and its entropic and entanglement diagnostics on representative steady states of the micromaser. For each configuration we compute the truncated field state ${\hat{\rho }}_{F}$, map it to the five-qubit register density operator ${\hat{\rho }}_{Q}$ in Gray and binary encodings, and evaluate the quantities introduced above.

In what follows we focus on three concrete steady states that are representative of the multi-block trapping, single-block trapping, and thermally connected regimes discussed in Section 5. All three are drawn from the same numerical database, computed in the Fock basis and then embedded into the five-qubit register using both the Gray and binary encoding. Table 1 summarizes their parameters and basic quantitative diagnostics, with numerical values taken directly from the dataset.

In addition to the scalar diagnostics listed in Table 1, we also provide multi-panel visualizations of the digitized register for the multi-block trapping configuration (case A), both in binary and Gray encodings and for two thermal occupations $n_{\mathrm{th}}=0$ and $n_{\mathrm{th}}=0.05$; see Figure 1, Figure 2, Figure 3 and Figure 4. These “QubitInfoPanel” plots combine field-level density-matrix information (density and phase plots in both Pegg–Barnett phase basis and Fock basis), photon-number statistics, and the register-level information graphs. In each information graph, the five qubits form the nodes and edges represent pairwise correlations. Edge thickness encodes the mutual information $I(b_{i}:b_{j})$, edge gray level encodes the bipartite negativity $N(b_{i}:b_{j})$, and each edge is labeled by $I(b_{i}:b_{j})$ with $N(b_{i}:b_{j})$ given in parentheses. Single-qubit entropies $S(b_{i})$ and other register-level diagnostics are collected in the outer panels of the multi-panel layout.

In cases A and B the interaction angle is tuned to a trapping value with $n_{q}+1=32$; case A uses q=4 (internal multi-block segmentation), whereas case B uses q=1 (single 32-dimensional trapping block). Case C corresponds to a hot cavity with no enforced trapping: here the trapping condition (4) is not imposed and the Liouvillian is thermally connected across the truncated space. In the numerical dataset this nontrapping regime is labelled by q=0 in the sense explained in Section 5.

### 6.1. Multi-Block Trapping with Weak Thermal Bridging

As a first example we consider a trapping configuration in which $n_{q}+1=32$ and the integer q=4 induces an internal segmentation of the truncated space into several trapping blocks. Atoms are prepared with positive inversion and full coherence (u=0.5,λ=1) and enter an almost empty cavity ($n_{\mathrm{th}}=0$), with a moderate pump parameter $N_{\mathrm{ex}}=500$ (case A in Table 1).

In this regime the steady-state photon-number distribution is confined to the lowest few Fock levels and exhibits clear plateaus associated with the trapping blocks. The mean photon number is modest, 〈n〉≈1.3, and the field entropy is extremely small, $S({\hat{\rho }}_{F})\approx 0.01$, indicating an almost pure steady state constrained by the trapping geometry. The photon-number statistics are characterized by the Mandel parameter $Q=\left(\langle n^{2}\rangle -{\langle n\rangle }^{2}-\langle n\rangle \right)/\langle n\rangle$ [33], which takes the value Q≈0.73 here, signaling super-Poissonian fluctuations. Many off-diagonal elements of the density matrix vanish exactly due to the block structure [1]. Single-qubit entropies in the Gray register are strongly inhomogeneous: their average is $\overset{——}{S(b_{i})}\approx 0.39$, but qubits whose Gray support overlaps the active blocks carry most of this entropy, while others remain nearly pure.

In the Gray-encoded register, the information graph reflects the segmentation of the trapping manifolds. A small number of qubit pairs carry most of the bipartite negativity, with $N_{\max}\approx 0.49$, forming a sparse pattern of strong edges, while many pairs are only weakly or not at all entangled. Three-tangles are nonzero on several triplets and reach a comparable maximum value, $\tau_{3}^{\max}\approx 0.49$, so that the strongest genuine tripartite correlations are of the same order as the strongest pairwise links. In this sense the multi-block regime is strongly entangled but geometry-constrained: the entanglement structure is largely captured by a few preferred sets of Gray bits dictated by the trapping geometry.

Turning on a small but finite thermal photon number ($0<n_{\mathrm{th}}\ll 1$) activates thermal bridging between trapping blocks without immediately destroying the internal structure of each block. For the same $\left(q,N_{\mathrm{ex}},u,\lambda \right)$ and Gray encoding the dataset shows that increasing $n_{\mathrm{th}}$ from 0 to 0.05 raises the field entropy from $S({\hat{\rho }}_{F})\approx 0.01$ to $S({\hat{\rho }}_{F})\approx 0.21$, while the mean photon number jumps to 〈n〉≈12. At the same time, the maximal pairwise negativity collapses from $N_{\max}\approx 0.49$ to $N_{\max}\approx 0.064$, whereas the maximal three-tangle decreases only to $\tau_{3}^{\max}\approx 0.36$ and then remains in the range 0.34–0.36 for $n_{\mathrm{th}}$ between 0.05 and 0.5; see Figure 5. Thermal jumps first populate the boundaries between trapping blocks and then gradually fill in neighboring blocks; on the information graph this manifests as the rapid weakening of the strongest two-qubit links while genuine tripartite correlations remain comparatively robust. The ratio $\tau_{3}^{\max}/N_{\max}$ thus increases sharply as soon as $n_{\mathrm{th}}$ is nonzero, indicating that the strongest genuinely tripartite structures are less sensitive to weak thermal bridging than the strongest two-qubit links.

A compact, multi-panel visualization of these two steady states in binary and Gray encoding is shown in Figure 1, Figure 2, Figure 3 and Figure 4. These “QubitInfoPanel” plots summarize, on a single page, the phase- and Fock-basis density matrices, photon-number distribution, and the register-level information graphs with both mutual information and negativity.

How to read Figure 1, Figure 2, Figure 3 and Figure 4. The central panels display the same steady state in the Pegg–Barnett phase basis and in the Fock basis (magnitude and argument), while the right panels translate the same state into a five-qubit information graph. In these graphs, thick edges indicate large mutual information (strong total correlations), and dark edges indicate large negativity (strong quantum correlations); thin, pale edges correspond to weak correlations. The main comparison across Figure 1, Figure 2, Figure 3 and Figure 4 is therefore the change from a sparse, strongly structured graph in the cold multi-block trapping regime to a much weaker (and more homogeneous) pairwise-entanglement pattern once even weak thermal bridging is enabled, and how Gray encoding makes the underlying trapping geometry appear as local features on the register.

Comparing Figure 1 and Figure 2 with Figure 3 and Figure 4 shows that the qualitative trends in single-qubit entropies, mutual informations, and pairwise negativities are closely similar in binary and Gray encodings for this multi-block trapping configuration. However, the Gray-encoded information graphs in Figure 3 and Figure 4 more directly reveal how trapping manifolds and thermal bridging act through local bit flips on low-order Gray bits: entropy is concentrated on a subset of Gray qubits whose support overlaps the active trapping blocks, and the weakening of the dominant two-qubit links with increasing $n_{\mathrm{th}}$ is immediately visible in the thinning and lightening of the corresponding edges and in the simultaneous reduction of the mutual-information and negativity values shown on those edges. Notably, the same thermal activation manifests differently on the register: in Gray encoding it primarily suppresses a small set of dominant local links tied to single-bit transitions, whereas in binary encoding it tends to redistribute correlations more broadly across qubit pairs, making the graph more homogeneous.

### 6.2. Single-Block Trapping and Broad Occupation

As a second example we choose a trapping angle for which $n_{q}+1=32$ but the truncated space forms a single invariant manifold, without internal block segmentation. Concretely, we consider q=1, $N_{\mathrm{ex}}=500$, u=0.5, $n_{\mathrm{th}}=0$, and λ=1 (case B in Table 1). Here, the same atomic preparation as in case A acts on a trapping geometry that does not split into smaller blocks.

In Fock space the steady-state photon-number distribution now occupies a broad band within the truncation, with mean photon number 〈n〉≈21.3 and an almost Poissonian Mandel parameter Q≈0. Despite this substantial occupation of higher Fock levels, the field entropy remains small, $S({\hat{\rho }}_{F})\approx 0.03$, reflecting a highly structured steady state. In Gray encoding, many single-step transitions n→n±1 are active and interfere in the steady state.

On the digitized register, the information graph is markedly different from the multi-block case. The average single-qubit entropy increases to $\overset{——}{S(b_{i})}\approx 0.55$, indicating that all five qubits are now strongly mixed in the Gray basis. Bipartite negativities are still peaked on a set of preferred edges, but their absolute scale is reduced, with $N_{\max}\approx 0.10$, and more pairs carry non-negligible entanglement. At the same time, the maximal three-tangle remains sizable, $\tau_{3}^{\max}\approx 0.31$, so that a significant fraction of the available entanglement “budget” is stored in genuine three-party correlations rather than in a few dominant pairwise links. Entropy and mutual-information profiles over the qubits reveal an emergent core–periphery structure: some qubits participate strongly in both entanglement and mutual information, while others are more weakly correlated but still far from pure.

This example shows that even at zero thermal occupation the removal of internal segmentation in the trapping manifold allows the same micromaser model to develop more collective, delocalized correlation structures on the digitized register. Compared to the multi-block case, pairwise entanglement is spread over a larger set of edges, and genuine tripartite contributions become more prominent relative to the strongest two-qubit links.

### 6.3. Thermally Connected Hot Cavity

As a third example we consider a regime in which the cavity is strongly coupled to a thermal reservoir with $n_{\mathrm{th}}\gg 1$, while atoms are prepared close to their ground state (u<0) with nonvanishing coherence. Thus, the atoms are near their ground state, whereas the cavity field is maintained in a highly excited, thermally populated regime. Specifically, we take the nontrapping label q=0, $N_{\mathrm{ex}}=2250$, u=−0.5, $n_{\mathrm{th}}=15$, λ=0.2 and a generic interaction angle Θ≈0.30 (case C in Table 1), so that no trapping condition is enforced. In the absence of atoms, the steady state would be a high-temperature thermal state; the atomic beam repeatedly perturbs and weakly cools this hot background.

The steady-state photon-number distribution in this regime resembles a truncated thermal distribution, with mean photon number 〈n〉≈4.6 and super-Poissonian Mandel parameter Q≈3.9. The field entropy is correspondingly high, $S({\hat{\rho }}_{F})\approx 1.52$, and the Liouvillian graph is strongly connected by thermal and Jaynes–Cummings transitions. The field thus provides a high-entropy resource on which the atomic map acts repeatedly, and the overall trend of $N_{\max}$ and $\tau_{3}^{\max}$ with increasing $n_{\mathrm{th}}$ in this regime is illustrated in Figure 6, where we use the same $\left(q,N_{\mathrm{ex}},u,\lambda ,\Theta \right)$ and vary $n_{\mathrm{th}}\in \left\{1,2,3,5,7,10,15,30,50\right\}$.

On the digitized register this regime is characterized by high single-qubit entropies, $\overset{——}{S(b_{i})}\approx 0.74$, and substantial mutual informations between several qubit pairs. Bipartite negativities are moderate, with $N_{\max}\approx 0.09$ at $n_{\mathrm{th}}=15$, reflecting the mixedness of the reduced two-qubit states, but the maximal three-tangle remains appreciable, $\tau_{3}^{\max}\approx 0.29$. As $n_{\mathrm{th}}$ increases from 1 to 50, the dataset shows that $N_{\max}$ decreases from about 0.11 to 0.05, whereas $\tau_{3}^{\max}$ decreases more slowly, from about 0.33 to 0.21.

From the register perspective, this trend addresses a question often associated with the semiclassical limit: in the present hot-cavity sweep, increasing $n_{\mathrm{th}}$ drives the field closer to a classical thermal mixture, i.e., a state that is more diagonal in the photon-number basis. Because register entanglement requires off-diagonal coherence of ${\hat{\rho }}_{F}$ under the chosen encoding, both $N_{\max}$ and $\tau_{3}^{\max}$ decrease as thermal mixing grows. (Mean photon number alone is not a sufficient classicality parameter; rather, the decay reflects loss of coherence and increased mixedness.)

In the semiclassical limit of simultaneously large mean photon number and strong thermal noise, the field density matrix approaches a diagonal thermal distribution in the truncated number basis; consequently, off-diagonal coherences vanish and the register entanglement measures (e.g., $N_{\max}$ and $\tau_{3}^{\max}$) are expected to decay to zero, consistent with the correspondence principle.

Correlations in this regime tend to be delocalized across more than two qubits: a description in terms of a few dominant two-qubit links is no longer adequate. The information graph takes on a more homogeneous appearance, with multiple edges and triplets participating in the correlation structure, consistent with the thermally connected Liouvillian geometry.

To make the difference in tripartite structure explicit, we compare the three-tangle on all ten triplets for a representative inverted, nearly cold configuration and a hot-cavity configuration with near-ground atoms in Figure 7. In the hot-cavity case, $\tau_{3}$ concentrates on an “information core” of low-order Gray bits, while in the inverted, cold case it is more evenly distributed across several triplets.

### 6.4. Entropy–Information Comparison Across Cases

The three representative configurations summarized in Table 1 and Figure 5, Figure 6 and Figure 7 allow a direct comparison of how entropy and correlation indicators are distributed.

In case A (multi-block trapping, weakly occupied), the mean photon number is small, 〈n〉≈1.3, and the field entropy $S({\hat{\rho }}_{F})\approx 0.01$ is far below the thermal value at the same intensity. The average single-qubit entropy is moderate, $\overset{——}{S(b_{i})}\approx 0.39$, and both $N_{\max}$ and $\tau_{3}^{\max}$ reach large values ≈0.49. Correlations are concentrated on a few Gray bits whose support overlaps the active trapping blocks, consistent with a sparse, strand-like information graph in which a small number of qubit pairs and triplets carry most of the entanglement.

In case B (single-block trapping, broadly occupied), the steady state populates most of the truncated ladder, with 〈n〉≈21.3 and an almost Poissonian Mandel parameter. The field entropy remains small, $S({\hat{\rho }}_{F})\approx 0.03$, but the register becomes more mixed, $\overset{——}{S(b_{i})}\approx 0.55$. The maximal three-tangle stays large, $\tau_{3}^{\max}\approx 0.31$, even though $N_{\max}$ drops to ≈0.10. This points to a redistribution of the entanglement “budget” from a few very strong two-qubit links (as in case A) towards more collectively shared tripartite correlations on a broader set of Gray bits.

Case C (hot cavity with near-ground atoms) combines a moderate mean photon number 〈n〉≈4.6 and strongly super-Poissonian statistics (Q≈3.9) with the largest field entropy in the set, $S({\hat{\rho }}_{F})\approx 1.52$. Single-qubit entropies are again high, $\overset{——}{S(b_{i})}\approx 0.74$, while $N_{\max}$ and $\tau_{3}^{\max}$ take comparable values around 0.09 and 0.29, respectively. Together with the triplet pattern in Figure 7, this indicates a high-entropy background on which correlations are spread over an “information core” of Gray bits rather than being localized on a small number of pairwise links.

Taken together, these trends demonstrate that field entropy, per-qubit entropy, and the topology of the information graph vary in a correlated but nontrivial way across the three Liouvillian geometries: segmented trapping, single-block trapping, and thermally connected hot cavity. The same micromaser model, when digitized into a five-qubit register, can thus realize qualitatively distinct patterns of how energy and information are distributed and shared across the qubits.

## 7. Discussion and Outlook

We have introduced a digitization-based analysis workflow for characterizing the entropy and correlation structure of a truncated bosonic field after embedding it into a small qubit register, and illustrated it on the steady state of a coherently pumped micromaser. By embedding the lowest 32 Fock levels of the cavity field into a five-qubit register using Gray or binary mappings, and by computing reduced entropies, mutual informations, bipartite negativities, and three-tangles on the register, we obtain an “information graph” description of the steady state that is directly expressed in qubit language. In particular, by using a Gray encoding that maps nearest-neighbor steps on the Fock ladder to single-bit flips on the register, we obtain a geometry-aware representation in which trapping manifolds and thermal bridges appear as structurally local features on the information graph, in contrast to the less transparent binary encoding.

A central role is played by the geometry of the micromaser Liouvillian in Fock space [1,4,5,6,7]. Trapping manifolds and thermal bridging determine which subsets of photon-number states are dynamically connected, and this structure is faithfully reflected in the information graph when Gray encoding is used. Multi-block trapping geometries give rise to sparse information graphs dominated by a few strong two-qubit links, with three-tangles of comparable magnitude. Trapping on a single 32-dimensional manifold or coupling to a thermally populated cavity, by contrast, enables broader occupation of the truncated Hilbert space and supports more delocalized correlation patterns in which genuine three-party contributions can become prominent.

From the perspective of entropy, these regimes differ not only in the overall field entropy but also in how entropy and mutual information are distributed over the qubits. Segmented trapping tends to concentrate entropy and correlations on the Gray bits whose support overlaps the active blocks, while other bits remain close to pure. Thermally connected regimes produce more homogeneous entropy profiles and spread mutual information over multiple qubits. The combination of entanglement monotones and entropic quantities thus provides a nuanced picture of how energy and information are stored and shared in the digitized field [26,27].

*Entanglement engineering by Liouvillian geometry*— These observations suggest viewing the micromaser as an entanglement-engineering platform for digitized fields. By tuning the trapping condition through *q* and Θ and by adjusting atomic inversion and thermal occupation, the same micromaser master equation can be steered between different information-graph topologies on the five-qubit register. In configurations with segmented trapping manifolds, the digitized field behaves as a set of weakly connected strands, while in connected geometries it exhibits more collectively shared correlations. Although the present study focuses on two- and three-party measures, the underlying mechanism is general: Liouvillian geometry and reservoir parameters sculpt the space of accessible correlations in the truncated field, and digitization makes this structure explicit at the qubit level. Within our numerical database, which covers several thousand parameter combinations spanning the three Liouvillian geometries, we have not found cases that contradict the qualitative patterns reported here.

There are several directions for further work.

Beyond single-mode micromasers, related questions of dissipation, thermalization and coherence generation in multimode nonlinear optical systems or in non-Markovian environments have become increasingly prominent (Refs. [20,21,22,23,24]). The present work isolates a solvable single-mode setting to make the Liouvillian-geometry-to-information-graph link explicit; extending the same digitization workflow to such multimode/non-Markovian settings is a natural direction for future study. First, a systematic exploration of digitized information graphs over a broader range of micromaser parameters, including different truncation dimensions and encodings, would help delineate which features are encoding-dependent and which are intrinsic to the underlying dynamics, in analogy with recent studies of field digitization in other contexts [17,18,19]. Second, extending the analysis to include measures of genuine multipartite entanglement beyond three parties, such as geometric entanglement or genuine multipartite negativity, could reveal higher-order structures not captured by negativity and three-tangle alone [27,31,32]. Third, applying the same digitization and information-graph framework to other driven-dissipative bosonic systems, such as multimode cavities, trapped ions, or superconducting resonators, would test its generality and potential use as a diagnostic tool in quantum simulation.

More broadly, the present work contributes to bridging the description of quantum fields in Fock space and the qubit-based language of quantum information. By treating digitization not just as a coding problem but as an opportunity to analyze entropy and correlations in a new representation, one gains access to structural insights that are difficult to obtain directly in the bosonic picture. We expect that such approaches will become increasingly relevant as quantum technologies bring bosonic and qubit degrees of freedom into the same architectures and as questions of entanglement structure and information flow move to the forefront of experimental design.

## Figures and Tables

**Table 1:** Representative micromaser steady states used as case studies (Gray encoding, Fock basis). For each case we list the pump and reservoir parameters $\left(N_{\mathrm{ex}},u,n_{\mathrm{th}}\right)$, the mean photon number 〈n〉, Mandel parameter *Q*, field entropy $S({\hat{\rho }}_{F})$, average single-qubit entropy $\overset{——}{S(b_{i})}=\frac{1}{5}\sum_{i}S\left(b_{i}\right)$, the maximum pairwise negativity $N_{\max}$ over all qubit pairs, and the maximum three-tangle $\tau_{3}^{\max}$ over all triplets. The three configurations correspond to multi-block trapping (A), single-block trapping (B), and a hot cavity with near-ground atoms (C).

Case	Type	$N_{\mathrm{ex}}$	*u*	$n_{\mathrm{th}}$	〈n〉	*Q*	$S({\hat{\rho }}_{F})$	$\overset{——}{S(b_{i})}$
A	Multi-block trapping, weakly occupied	500	0.5	0	1.27	0.73	0.01	0.39
B	Single-block trapping, broadly occupied	500	0.5	0	21.29	0.00	0.03	0.55
C	Hot cavity with near-ground atoms	2250	−0.5	15	4.55	3.87	1.52	0.74
					$N_{\max}$		$\tau_{3}^{\max}$	
A					0.49		0.49	
B					0.10		0.31	
C					0.09		0.29	

## Data Availability

The original contributions presented in this study are included in the article. Further inquiries can be directed to the corresponding author.
